# Pain in a chromium-allergic patient with total knee arthroplasty: disappearance of symptoms after revision with a special surface-coated TKA ­— a case report

**DOI:** 10.3109/17453674.2011.579521

**Published:** 2011-07-08

**Authors:** Marc Thomsen, Matthias Rozak, Peter Thomas

**Affiliations:** ^1^Department of Orthopaedics, DRK-Klinik, Baden-Baden; ^2^Department of Dermatology, Ludwig-Maximilians-Universität München, Munich, Germany

In 2005, a 60-year-old woman suffering from osteoarthritis received a total knee replacement (TKA) (e.motion, BBraun Aesculap, Tuttlingen, Germany) in another hospital. The prosthesis was implanted using antibiotic-loaded cement. Postoperatively, she suffered reduced mobility (E/F 0/10/60°) and her knee pain did not get better. In 2006, since the pain continued, a cemented retropatellar replacement was implanted. Radiological examination did not reveal any sign of a mechanical complication, but the pain still persisted and the patient was admitted to our hospital. Now, she complained of partly eczematous reactions (local itching, partial oozing, eczematous rashes), which appeared about half a year after the primary surgery (Figure 1). Blood counts including C-reactive protein test and bacteriological tests after joint aspiration virtually excluded a low-grade infection. A lymphocyte transformation test showed no increased values for metal ions (chromium, cobalt, nickel).

**Figure 1.**
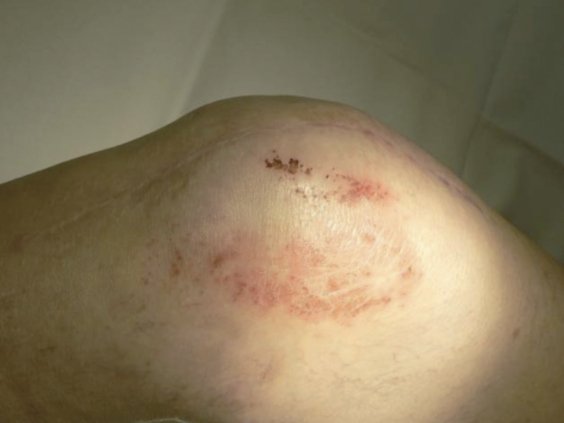
Eczematous reaction after TKA.

Thus, in February 2009, the implant was replaced by a device that was geometrically identical to the initial prosthesis but was covered with an anti-allergic ZrN multilayer coating on the standard CoCr^29^Mo^6^ implant (Figure 2). It consists of 7 layers, a very hard shielding layer, ZrN, 5 intermediate layers which gradiently applied bridge the differences in hardness and residual stress between softer base material and hard top coating and a Cr bond coating which ensures adherence of the coating. The interfaces between the layers constitute an additional diffusion barrier against ions from the base material (Reich et al. 2010).

**Figure 2.**
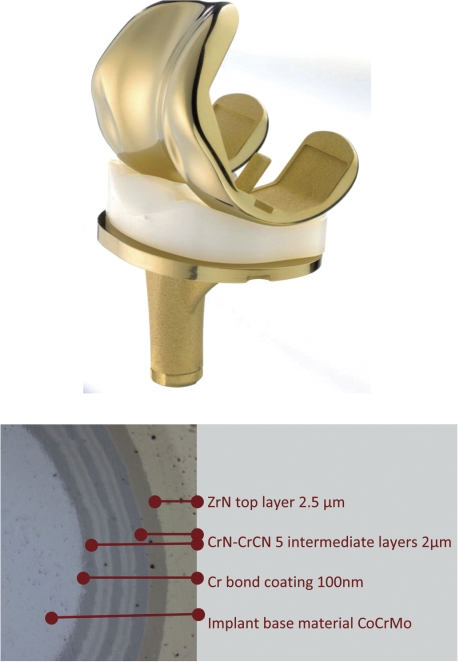
ZrN-CrN-CrCN multilayer coating.

The wound healed without complications and the eczema disappeared. Furthermore, at the last follow-up session in August 2010, 18 months after the revision, the mobility of the patient was excellent, with values of E/F 0/0/115°. The knee pain had disappeared.

## Discussion

There is evidence that the risk of complications after arthroplasty in metal-allergic patients is low (Thyssen et al. 2009). Even so, there have been prospective studies showing that patients with failed implants have a higher incidence of metal allergy (Hallab et al. 2001, Thomas et al. 2009).

Since the 1980s, several reports associated local eczema or erysipelas—e.g. erythema and swelling—with metal allergy (Jäger and Balda 1979, Schuh et al. 2008). This was observed particularly in patients with extremity osteosynthesis that was sensitive to nickel or cobalt (Schuh et al. 2008) and in patients with cerclage after sternotomy. A case report pointed to potential hyper-reactivity to metal close to the skin (Thomas et al. 2006). In a recent study conducted on 233 patients in a hospital specializing in implant allergies, it was found that 75% of patients with complications were arthroplasty patients. The symptoms were pain (68%), local swelling (42%), erythema (33%), loosening (20%), and eczema (18%) (Thomas et al. 2009). Patients rarely had vasculitic or urticarial reactions.

The prevalence of allergic reactions after knee or hip replacement is unknown. No causal relationship has been shown between the frequency of cutaneous metal allergies in the population and the rates of complications in orthopedic patients caused by metal allergy. Schuh et al. (2008) reported 12 cases of allergy to nickel, four to cobalt, and one case each to chromium and benzoyl peroxide in 300 arthroplasty patients, but only 1 patient was symptomatic. However, metal sensitivity was found in two-thirds of 16 cases with failed metal-on-metal hip arthroplasty and peri-implant lymphocytic inflammation (Thomas et al. 2009).

Unfortunately, there is no standard diagnostic procedure for investigation of a suspected implant allergy. However, the German Orthopaedic and Allergological Societies have recently issued an interdisciplinary statement that summarizes the current knowledge on the subject and also serves as a guideline for the treatment options of potentially allergic patients (Thomas et al. 2008). As in our case, anamnesis is the first step in diagnosing metal implant allergy. A pre-existing or new contact allergy, or eczema, after implantation raises strong suspicion, and it must be confirmed by the clinical picture (Hallab et al. 2005).

In our opinion, a preoperative biopsy must be carried out to reliably exclude a low-grade infection. Additionally, an ECT allergy test and, if necessary, a lymphocyte transformation test (LTT) can confirm the diagnosis. However, LTT lacks specificity and should only be used as a complement to other tests (Schuh et al. 2008). If the prosthesis is cemented, allergic reaction to cement components could be the reason for aseptic loosening and often there is a link to a metal hypersensitivity reaction (Raimondi and Pietrabissa 2000).

After these investigations, allergy should be the working diagnosis when no other diagnosis seems likely.

In cases of metal allergy, femoral and tibial components made of CoCrMo or a titanium alloy covered with a PVD layer of titanium nitride or titanium niobium nitride are increasingly being used to reduce ion release into the periprosthetic tissue (Thull et al. 1995, Reich et al. 2010). Single-layer coated implants, as standard or customized versions, are most often being offered. Ceramic single-layer coatings exhibit a high degree of hardness and good wear resistance, but can chip off from the softer base material in rare cases (Hendry and Pilliar 2001), inducing third-body wear. Oxinium prostheses (without any coating, but with a special surface treatment) (Bader et al. 2008) are usable alternatives. To minimize the risk of layer wear due to an excessive difference in hardness and residual stress gradients, a multilayer approach to covering CoCrMo implants has been developed that causes only minor tensile stress within the layer, and it has been shown to avoid delamination in an in vitro set-up (Reich et al. 2010). In our patient, the new multilayer-coated implant has shown excellent results after 18 months. Further studies are necessary to prove the good clinical outcome on a larger scale. A prospective randomized study is underway to investigate the metal ion concentrations of this new implant compared to standard uncoated CoCrMo implants (Lützner et al. 2009).
